# Relationship between frailty and depressive symptoms in older adults: role of activities of daily living and sleep duration

**DOI:** 10.3389/fmed.2024.1416173

**Published:** 2024-06-26

**Authors:** Wei Song, Manyu Liu, Ting Ye, Dong Wang, Quan Yuan, Fen Li, Qiushi Wang, Yana Ma

**Affiliations:** ^1^School of Public Health, Jiangsu Key Laboratory of Preventive and Translational Medicine for Geriatric Diseases, MOE Key Laboratory of Geriatric Diseases and Immunology, Suzhou Medical College of Soochow University, Suzhou, Jiangsu, China; ^2^Suzhou Xiangcheng Second People’s Hospital, Suzhou, Jiangsu, China

**Keywords:** frailty, depressive symptoms, activities of daily living, moderated mediation effect, older adults

## Abstract

**Introduction:**

Previous studies have demonstrated that frailty is associated with depressive symptoms among older people and significantly increase the risk of difficulty in activities of daily living (ADL). However, uncertainties remain regarding the mechanisms behind such relationship. The aim of this study was to investigate the mediating effect of ADL in the relationship between frailty and depressive symptoms among older adults in China, and to explore to what extend sleep duration moderated the association between ADL and depressive symptoms.

**Methods:**

In this study, we carried out cross-sectional descriptive analysis and 1,429 participants were included in the analysis. A survey was conducted using questionnaires and instruments measuring frailty, depressive symptoms, ADL and sleep duration. Bootstrap analyses served to explore the impact of ADL in mediating frailty and depressed symptoms, as well as the effect of sleep duration in moderating ADL and depressive symptoms.

**Results:**

Compared to the robust group, the mediating effects of ADL between frailty and depressive symptoms were significant in the prefrail group and the frail group. The interaction term between sleep duration and ADL was significantly presented in the regression on depressive symptoms. Specifically, the Johnson–Neyman technique determined a range from 8.31 to 10.19 h for sleep duration, within which the detrimental effect of frailty on depressive symptoms was offset.

**Conclusion:**

Sleep duration moderated the indirect effect of ADL on the association between frailty and depressive symptoms. This provides support for unraveling the underlying mechanism of the association between frailty and depressive symptoms. Encouraging older adults to enhance ADL and obtain appropriate sleep duration might improve depressive symptoms for older adults with frailty and prefrailty.

## 1 Introduction

The global share of population aged 65 years or above is projected to rise from 10% in 2022 to 16% in 2050 (1), which reflects the severity of the world’s population aging. It is well known that population aging gives rise to many geriatric phenomena, of which frailty is the most salient (2). Frailty is defined as a biologic syndrome marked by decreasing physical power, endurance, and impaired physiological function, making older people more vulnerable to developing reliance and death (3). Frailty occurs with age and carries a high risk of multiple negative health outcomes, such as depressive symptoms, falls, difficulty in ADL, and disability (4–7). With the aging population growing, the link between frailty and these negative outcomes may pose significant challenges to the capacity of healthcare systems (8), especially in China which has the largest older population in the world (190.64 million people aged 65 and above in 2020, accounting for 13.5% of the total population) (9).

Depressive disorder is assumed to the most common psychological problem among older people and has become one of the leading risk factors for disability, greatly contributing to the worldwide disease burden (10). A meta-analysis involving 57,486 subjects found a high prevalence rate of depression among older people of 31.74% in the world (11). Similar to frailty, depressive symptoms also boost susceptibility to unfavorable outcomes with increasing age (12). According to previous studies, frailty has been proven to be the predicter not only of the onset but also of the persistence of depressive symptoms in older age (13, 14). Furthermore, a reciprocal interaction between frailty and depressive symptoms has also been demonstrated (15). However, despite of the fact that many studies focused on the impact of frailty on depressive symptoms, question about the underlying mechanisms is still unanswered.

Activities of daily living (ADL) refer to the essential abilities required to handle basic physical requirements (16). Individuals with declining instrumental activities of daily living (IADL) were prone to have weaker basic activities of daily living (BADL) and had less capacity to carry out high-level regular activities and social life (17). As an important indicator of geriatric physical function, ADL has been reported to be closely associated with both frailty and depressive symptoms (6, 12). According to a systematic review, the risk of developing, worsening, and combining ADL and IADL disability was approximately two times or higher for people classified as frail compared to those classified as non-frail (18). This review also suggested these risks were apparent in prefrail individuals but to a lower extent.

There were evidences for the negative impact of ADL functioning on depressive symptoms (19, 20). Meanwhile, a cross-sectional study demonstrated that disabilities in both physical self-care and IADL were positively associated with depressive symptoms, accompanied by gender differences (21). The mobility of older adults tends to decline over the life course as they age, making plenty of daily activities are tough to carry out on their own (22). Then reduced mobility may increase older adults’ fear of ageing and death and induce depressive symptoms (23). Therefore, we hypothesize that frailty may have an indirect influence on depressive symptoms in older adults through the mediating effect of ADL.

Depressive symptoms often occur in conjunction with sleep disorders as well (24). In addition, sleep duration was significantly associated with an increased risk of depressive symptoms (25). Studies have showed that in comparison with normal sleep duration (7–9 h for adults), either short and long sleep duration had potential to raise the risk of depression (26). Sufficient sleep duration can assist older people in performing both physical and psychological functions adequately (27). Thus, sleep duration may play a moderating role between ADL and depressive symptoms.

Our study is aimed at exploring the relationship between frailty and depressive symptoms, as well as clarting whether ADL mediates the impact of frailty on depressive symptoms with sleep duration acting as a moderator.

## 2 Methods

### 2.1 Study design and participants

This population-based cross-sectional study was conducted in Suzhou, Jiangsu Province, China, from September 2022 to December 2022. Participants were recruited at a general hospital in Xiangcheng District through convenience sampling. The purpose of this study and other relevant information was explained to the potential participants prior to the study enrollment. All participants were voluntary to be involved in this study and provided written informed consent. Trained researchers collected data through face-to-face interviews and physical tests. The study obtained approval from the ethics committee of Zhong Shan Hospital [NO. B2020-201 (3)] and followed the principles outlined in the Declaration of Helsinki. The eligible participants consisted of elderly people aged above 65 years old, able to perform physical tests, with an absence of cognitive impairments and serious or life-threatening conditions. Finally, a sample of 1,574 eligible persons was obtained, and of these, 145 individuals with missing information were filtered out, leaving 1,429 persons who entered the current analysis.

### 2.2 Measures

#### 2.2.1 Frailty

Frailty was assessed based on a phenotype model by Fried, a widely used screening scale in the field of geriatrics (28). There are 5 components involved in frailty, which are weight loss, exhaustion, low physical activity, slow pace and weakness. Details of assessment criteria for frailty we used are available as Supplementary Table S1 (29). Each compliant indicator represents one point, and a higher score indicates a worse state of frailty. Participants scoring 0, 1 or 2, and 3 or more were diagnosed as robust, prefrail, frail, respectively.

#### 2.2.2 Depressive symptoms

Depressive symptoms were assessed using the Chinese version of Patient Health Questionnaire-9 (30), which has been proven to be an effective screening questionnaire for depression in China (31). The PHQ-9 has 9 items and all items are rated on a four-point Likert-type scale. Participants rated frequency of experiencing each symptom from 0 (not at all) to 3 (nearly every day) with the total scores ranging from 0 to 27. In this study, the Cronbach’s alpha for the scale was 0.784.

#### 2.2.3 Activities of daily living

Disability in activity of daily living in older adults were evaluated by the Activity of Daily Living Scale (ADLS) with 2 subscales within Lawton and Brody (32). One of the subscales is the Physical Self-Maintenance Scale (PSMS) including 6 items about toileting, feeding, dressing, grooming, physical ambulation, and bathing. The other subscale is the Instrumental Activities of Daily Living Scale (IADLS), which consists of 8 items. All the ADLS items are rated through a four-level scoring method, thus the total score ranges from 14 to 56. The higher the score, the less capable the participant is of achieving daily activities independently. A total score of 14 means no incapacity, two and more items ≥3 points or a total score of 22 and higher is considered as functional impairment. In the present study, the Cronbach’s was 0.755 for the PSMS and 0.739 for the IADLS.

#### 2.2.4 Moderator and covariates

Sleep duration was taken into account in the analysis to explore their moderating effects. The measurement of sleep duration was relied on the question “How many hours of sleep do you get every night averagely?.” Participants were also asked about their usual sleep onset and wake-up time to get an accurate sense of how long they slept. Covariates were split into sociodemographic variables and health-related variables. Sociodemographic features contained age, sex, education level, marital status, and annual income. Health-related features included smoking status, drinking conditions, weekly exercise, chronic disease conditions, number of medications, falls experience in the last year, grip strength, gait speed, body mass index (BMI).

### 2.3 Statistical analysis

**Figure 1:**
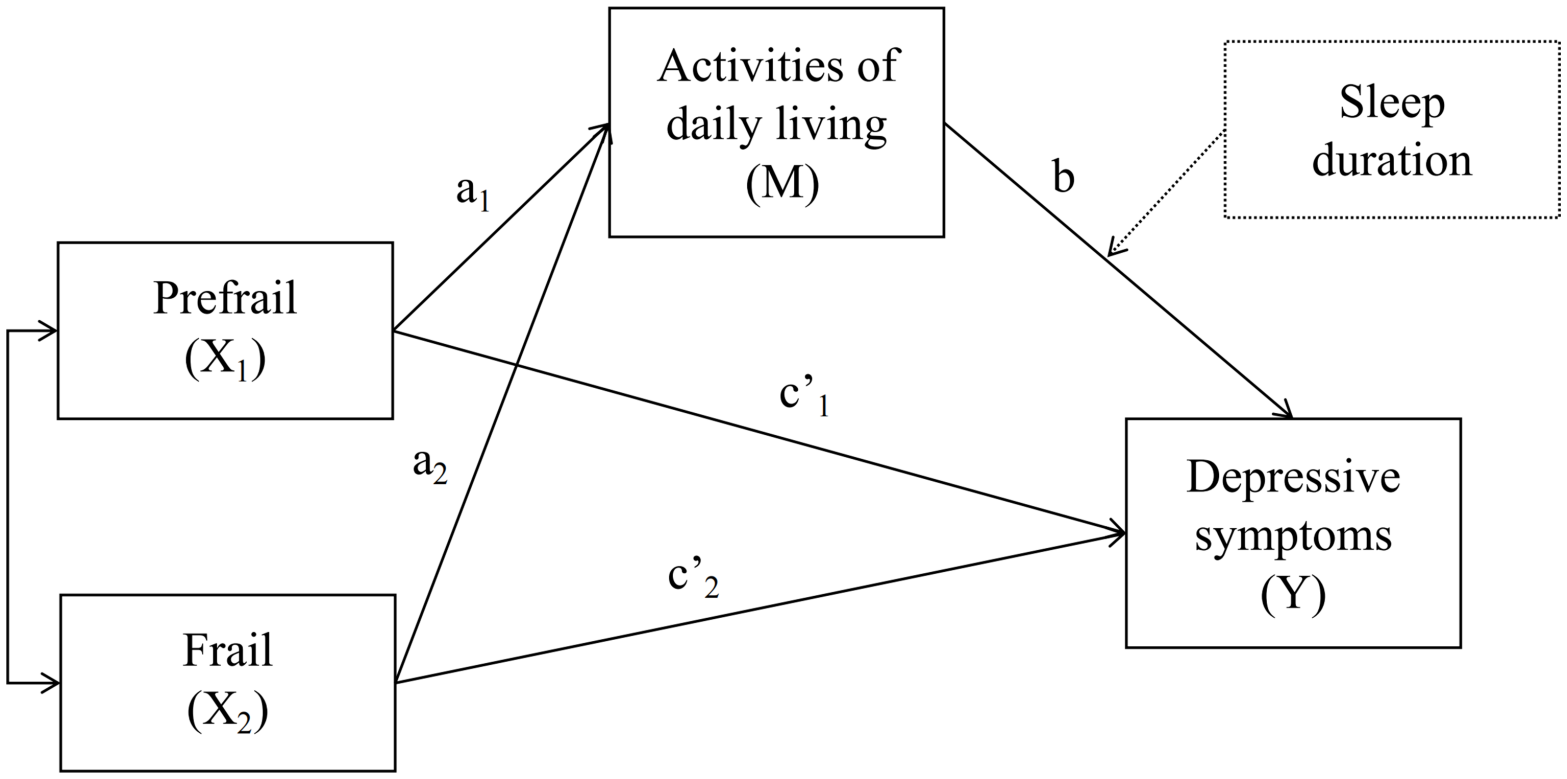
The proposed moderated mediation model. The independent variable—frailty (X) has three levels, which are robust (X0), prefrail (X1), frail (X2). a1b is the indirect effect on Y via M of being in the X1 group relative to the X0 group, and c’1 represents the direct effect of being in the X1 group on Y relative to the X0 group; a2b is the indirect effect on Y via M of being in the X2 group relative to the X0 group, and c’2 represents the direct effect of being in the X2 group on Y relative to the X0 group. The dashed lines indicate the subsequent introduction of moderating variable—sleep duration.

SPSS version 27.0 software for Windows (IBM Corp) was used for carrying out all the statistical analyses. The statistically significant results were detected at a minimum of *p* value <0.05. Continuous variables and discrete variables were described using means (standard deviations) and frequencies (percentages) respectively. Pearson correlation coefficients between all the study variables were calculated (results are shown in Supplementary Table S2). The covariates significantly related to the outcome variable (depressive symptoms) were controlled in this study. The mediation model and moderated mediation model were tested using the SPSS PROCESS version 4.0 macro. Considering the independent variable (frailty) was viewed as a trichotomous variable, the method about relative indirect, direct, and total effect developed by Hayes and Preacher (33) was adopted (See Figure 1 for details on the moderated mediation model framework). First, dummy coding was used in the independent variable (frailty) with the robust group functioning as the reference group. Second, whether ADL mediated the association between frailty and depressive symptoms was tested. In this study, the indirect effect consisted of two relative indirect effects. ADL can be considered as the mediator between frailty and depressive symptoms if there was at least one significant relative indirect effect (33). Third, sleep duration was added as the moderator between ADL and depressive symptoms. The Johnson - Neyman technique was used to explore the boundary values of the moderating effect (34). The bootstrap 95% CI for mediating and moderating effects were estimated based on 5,000 repeated samples. The significance of the bootstrap 95% CI was confirmed with the confidence interval not including zero.

## 3 Results

### 3.1 Characteristics of participants

Table 1 shows the sociodemographic and health-related features of participants. The mean age of all the participants was 71.69 (SD = 4.94) years, 743 (51.99%) were women, and 613 (42.90%) participants had no formal school. The majority of participants were married (85.30%) and had annual income of less than 30,000 RMB (87.26%). The proportions of smoking and drinking participants were 25.68 and 18.05%, respectively. More than half of the participants exercised every day of the week (57.45%). Approximately 63.47% of the participants suffered from one or more chronic diseases, and only 9.52% took three or more medications a day. The mean BMI was 24.37 (SD = 3.32) kg/m^2^ and mean sleep duration was 7.22 (SD = 1.78) hours. Their average score was 2.16 (SD = 2.83) for depressive symptoms, 14.68 (SD = 2.14) for ADL and 1.21 (SD = 0.93) for frailty. Of the 1,429 participants, 18.33% were considered as having ADL disability and 9.03% as having frailty.

**Table 1:** Characteristics of the study participants.

Variables	Mean ± SD / *n* (%)	Variables	Mean ± SD / *n* (%)
Age(years)	71.69 ± 4.94	Number of chronic diseases	
Sex		0	522 (36.53)
Male	743 (51.99)	1	678 (47.45)
Female	686 (48.01)	2	197 (13.79)
Education level		≥3	32 (2.24)
No formal school	613 (42.90)	Number of medications	
Primary school	586 (41.01)	0	500 (34.99)
Middle school	204 (14.28)	1–2	793 (55.49)
High school or above	26 (1.82)	≥3	136 (9.52)
Marital status		Falls in the last year	
Single/divorced/widowed	210 (14.70)	No	1,258 (88.03)
Married	1,219 (85.30)	Yes	171 (11.97)
Annual income, RMB		Grip strength, kg	24.49 ± 9.54
<30000^*^	1,247 (87.26)	Gait speed, km/h	2.72 ± 0.59
≥30,000	182 (12.74)	BMI, kg/m^2^	24.37 ± 3.32
Smoking		Depressive symptoms score	2.16 ± 2.83
No	1,062 (74.32)	ADL score	14.68 ± 2.14
Yes	367 (25.68)	ADL disability	
Drinking		No	1,167 (81.67)
No	1,171 (81.95)	Yes	262 (18.33)
Yes	258 (18.05)	Sleep duration, hour	7.22 ± 1.78
Weekly exercise		Frailty score (0–5)	1.21 ± 0.93
Never	414 (28.97)	Frailty status	
1–3 times	127 (8.89)	Robust	352 (24.63)
4–6 times	67 (4.69)	Prefrail	948 (66.34)
Every day	821 (57.45)	Frail	129 (9.03)

ADL, activities of daily living; BMI, body mass index; SD, standard deviation. *3,000 RMB = approximately 4,330 USD.

### 3.2 Mediation effect and moderated mediation effect of frailty on depressive symptoms

Table 2 presents hierarchical regression analysis results and Table 3 shows the bootstrap test results. Frailty had a significant predictive effect on ADL, which meant compared with the robust group, the prefrail (β = 0.368, *p*<0.01) and frail group (β = 1.349, *p* <0.001) were both positively associated with ADL. However, compared with the robust group, the frail group (β = 0.867, *p* <0.01) was positively associated with depressive symptoms while the prefrail group (β = 0.015, *p* >0.05) showed no association with depressive symptoms. ADL (β = 0.179, *p* <0.01) also had a significantly predictive effect on depressive symptoms. Meanwhile, with the robust group as the reference level, the 95% bootstrap confidence interval for the relative indirect effect was [0.024, 0.123] for the prefrail group and [0.073, 0.501] for the frail group, neither of which included 0, indicating a significant relative mediating effect. However, the relative total effect and relative direct effect on depressive symptoms were significant in the frail group, but not in the prefrail group. For the relative indirect effect as a proportion of the relative total effect, it was 21.75% in the frail group.

**Table 2:** Linear regression models for the mediation effect and moderated mediation effect of frailty on depressive symptoms.

Predictors	Outcome variables							
	ADL		Depressive symptoms		ADL (moderated by sleep duration)		Depressive symptoms (moderated by sleep duration)	
	β	*t*	β	*t*	β	*t*	β	*t*
Sex	0.588	4.412^***^	0.476	2.675^**^	0.588	4.411^***^	0.374	2.188^*^
Smoking	−0.170	−1.109	0.077	0.378	−0.170	−1.109	0.066	0.342
Weekly exercise	0.013	0.313	−0.128	−2.270^*^	0.013	0.313	−0.118	−2.201^*^
Number of chronic diseases	0.031	0.416	0.261	2.648^**^	0.031	0.416	0.244	2.589^**^
Prefrail	0.368	2.784^**^	0.015	0.084	0.368	2.784^**^	0.127	0.754
Frail	1.349	6.117^***^	0.867	2.923^**^	1.349	6.117^***^	0.869	3.055^**^
ADL			0.179	5.074^***^			0.968	6.827^***^
Sleep duration							1.193	4.227^***^
ADL × Sleep duration							−0.107	−5.688^***^
*R^2^*	0.053		0.050		0.053		0.131	
*F*	13.2466^***^		10.710^***^		13.247^***^		23.782^***^	

ADL, activities of daily living. All the models were controlled for sex, smoking, weekly exercise and number of chronic diseases. ^*^p < 0.05, ^**^p < 0.01, ^***^p < 0.001.

**Table 3:** Bootstrap tests for the mediation effect and moderated mediation effects of frailty on depressive symptoms.

	Type of effect	Sleep duration	Effect size	Boot SE	Boot ULCI	Boot LLCI
Prefrail	Total effect	—	0.081	1.163	−0.246	0.395
	Direct effect	—	0.015	0.162	−0.306	0.328
	Indirect effect	—	0.066	0.026	0.024	0.123
Frail	Total effect	—	1.108	0.351	0.433	1.809
	Direct effect	—	0.867	0.331	0.226	1.524
	Indirect effect	—	0.241	0.110	0.073	0.501
Pre-frail (moderated by sleep duration)	Direct effect	—	0.127	0.154	−0.182	0.424
	Indirect effect	M-1SD	0.141	0.041	0.070	0.230
		M	0.071	0.024	0.032	0.124
		M + 1SD	0.001	0.021	−0.040	0.043
Frail (moderated by sleep duration)	Direct effect	—	0.869	0.313	0.265	1.493
	Indirect effect	M-1SD	0.518	0.182	0.225	0.927
		M	0.261	0.092	0.112	0.466
		M + 1SD	0.004	0.078	−0.176	0.142

Boot, bootstrap; SE, standard error; LLCI, lower limit of confidence interval; ULCI, upper limit of confidence interval; M, mean; SD, standard deviation.

**Figure 2:**
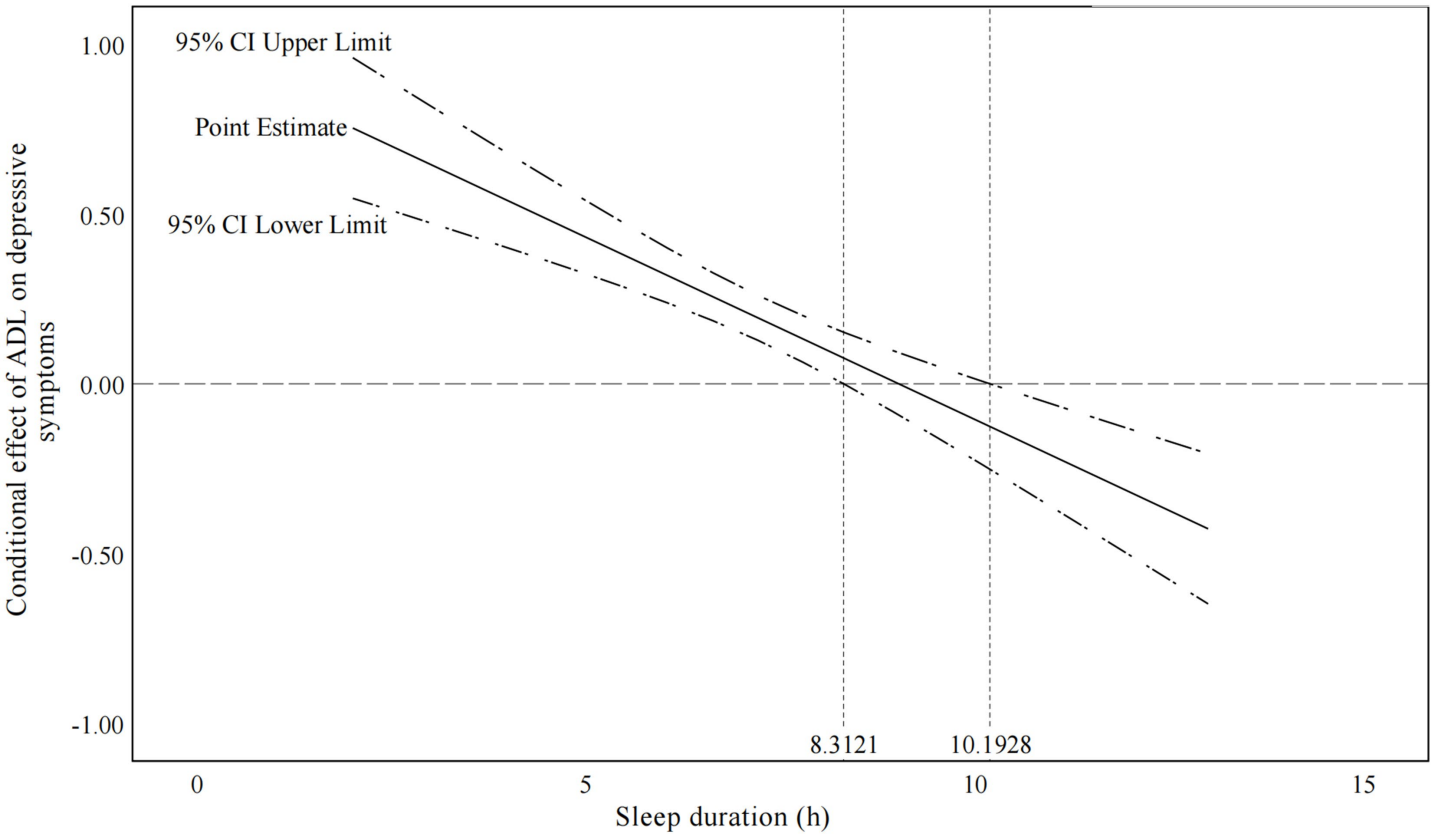
Conditional effect of ADL on depressive symptoms across the range of sleep duration. When the sleep duration is outside the interval [8.31, 10.19], the slope of ADL on depressive symptoms dependency is significant. ADL, activities of daily living.

Moderated mediation models in Table 2 reflected that sleep duration (β = 1.193, *p* <0.001) and the interaction term between ADL and sleep duration (β = −0.107, *p* <0.001) significantly predicted depressive symptoms. This showed the moderating role which sleep duration played in the relationship between ADL and depressive symptoms. As shown in Table 3, the predictive effect of ADL on depressive symptoms was stronger for participants with shorter sleep duration (M-SD) than for participants with average sleep duration (M). Moreover, for the participants with longer sleep duration (M + SD), ADL was unable to mediate the relationships between frailty and depressive symptoms. The Johnson–Neyman plot further indicated that the effect of ADL on depressive symptoms was completely offset by sleep duration above the threshold of 8.31 h and also below the threshold of 10.19 (Figure 2).

## 4 Discussion

To the best of our knowledge, this is the first study to establish a moderated mediation model with ADL serving as a mediator in the relationship between frailty and depressive symptoms, and sleep duration serving as a moderator in the path between ADL and depressive symptoms. We also revealed that with increasing sleep duration, the change in the effect of frailty on depressive symptoms tends to decrease to zero and then increase. In other words, only adequate and appropriate sleep duration was likely to counteract the deleterious effects of frailty on depressive symptoms. Noteworthy, the variable of frailty in this study was included in the analysis as categorical variable.

We found that frailty is positively associated with an increased risk of developing depressive symptoms, which is consistent with prior studies (14, 35). To elaborate further, depressive symptoms are more severe in frail and prefrail older people. The possible explanation is that due to physical impairment, frail individuals are usually unable to participate more in social activities, resulting in more depressive symptoms (36). Moreover, low-grade inflammation caused by frailty may increase susceptibility to depressive symptoms through activating the hypothalamus-pituitary-adrenocortical axis (37). There is a consensus that the process of frailty is potentially reversible, with prefrailty being capable of reversing to a robust state or downgrading to a frail stage (38). Meanwhile, Huang et al. (39) has showed that depressive disorder was the most common mood disorder in China (lifetime prevalence of 6.8%), and its risk occurring at middle and old age is apparently higher than other age. Due to disease burden and high prevalence, it is crucial to put frailty conditions into consideration for interventions of depressive symptoms.

The finding that elevated level of ADL helped to amelioration of frailty complements previous studies (18, 28). On the basis of CHARLS survey, Zhang et al. (7) illustrated that frail older people have a higher risk of ADL and IADL difficulties in the future. Meanwhile, depressive symptoms are often accompanied by ADL impairment among older people (40). He et al. (19) also indicated that ADL impairment was connected to an elevated likelihood of depressive symptoms. It is possible that limitations in daily activities and physical function cause older adults to lose their independence (22), causing them to turn to others for more support with feelings of guilt and uselessness (41), which may greatly contribute to the emergence or worsening of depressive symptoms.

Our study revealed the mediating role of ADL between frailty and depressive symptoms. Older adults with poorer frailty state are less able to conduct daily activities, leading to an elevated severity of depressive symptoms. This implies that strengthening ADL is vital to alleviating depressive symptoms in seniors, particularly prefrail and frail ones. Physical exercise has been proven to be a useful alternative to antidepressants in the treatment of geriatric depression (42), as well as to facilitate increased gait speed, better balances and improves ADL performance in frail older adults (43). Additionally, Catalan-Matamoros et al. (44) suggested older adults with depressive symptoms to find the suitable form of exercise and set practical goals. Thus, older adults ought to be encouraged to get appropriately involved in physical exercise fit for them.

It is worth noting that the relative total and relative direct effect of frailty on depressive symptoms in this study is present only in the frail group, but not in the prefrail group, under the reference level of the robust group. This provides two indications: (1) ADL partly mediates the association between frailty and depressive symptoms for frail older adults (relative mediating rate of 21.75%); (2) in view of discussion by Rucker et al. (45), we refrain from using the term ‘full mediation’ and interpret the direct effect as frailty affecting depressive symptoms mainly through ADL for prefrail older adults. Fernández-Garrido et al. (46) wrote the incidence of prefrailty was substantially greater than the comparatively low prevalence of frailty among older adults (7–12% of the population over 65 and roughly 25% of the population over 85), which is in line with our results. Gary (47) also found medium-intensity exercise yielded a positive effect on falls and physical performance in prefrail but not in frail older adults. Therefore, more attention possibly should be paid to prefrailty to bring about a real state reversal.

We further found that sleep duration moderated the relationship between ADL and depressive symptoms. The indirect effect is stronger in individuals who slept for shorter or longer periods of time. Obtaining approximately 8 to 10 h of sleep per night can even entirely offset the negative effects of decreased ADL on depressive symptoms. It can be speculated that older adults with long sleep duration may elevate their inflammatory markers, leading to an influence on the impairment of their cognition and motion, thus increasing the possibility of ADL disability and ultimately inducing depressive symptoms (48). Luo et al. (49) argued that short and long sleep durations amplified the risk of hypertension among Chinese adults, which might result in cardiovascular diseases that impair senior people’s capacity for daily activities. There is currently few research on the optimum duration of sleep for older adults opposed to the general population. Sleep duration of 6–8 h was connected with the lowest risk of death in a study comprising 116,632 adults, while the optimal amount of sleep duration was roughly 7 h (50). However, one recent study supporting our findings has also highlighted that two more hours of sleep may be advantageous for older adults’ daily activities (48). In addition, the impact of baseline and deteriorating frailty states on the increased severity of depression symptoms can be exacerbated by short sleep duration (51), which partly validated our results. A plausible explanation may be that older adults require more nocturnal rest to sustain strength and regain functions. To fully understand and elucidate the effect of sleep duration in geriatric health, further study is necessary. Taking together, frailty may cause more interference in older adults with shorter or longer sleep durations through ADL and eventually increase the risk of depressive symptoms.

Significant implications from this study are embodied in the management and prevention of the influence of frailty on depressive symptoms. For health care physicians and staff, we suggest that when diagnosing and managing frail older persons, greater focus be devoted to the contemporaneous emergence of depressive symptoms. Besides, older individuals should be motivated to engage in ADL-improving behaviors in order to alleviate their depressive symptoms. In particular, interventions targeted on sleep duration deserve more priority to mitigate the effects of ADL on mental well-being. Nevertheless, there are several limitations in this study. First, given our study was cross-sectional design, we could not offer solid proof for causal interpretation of observed relationships. Future longitudinal studies are strongly warranted to validate causal associations. Second, the information collected was self - reported by the participants, which could bring about recall bias. We have tried to minimize bias through application of objective indicators and professional investigators. Third, the sample from a Chinese city hinders contributions to generalization of our study. A population balance between urban and rural areas should be taken into account when it comes to the scope of the study subjects. Finally, even though models incorporated an extensive range of factors impacting depressive symptoms, residual confounding could remain due to neglected confounders. Further research should widely select potential confounders, including medical background and essential signs (such as blood pressure, blood sugar, etc.), to acquire a more thorough evaluation.

## 5 Conclusion

This study may provide support for unraveling the underlying mechanism of the association between frailty and depressive symptoms and developing an efficient approach to mitigate depressive symptoms among older adults. In accordance with this study, ADL mediated the relationship between frailty and depressive symptoms, with sleep duration functioning as a moderator between ADL and depressive symptoms. Specifically, frail and prefrail older adults may be more likely to suffer from depressive symptoms through limited ADL than robust ones. Meanwhile, enough sleep duration may diminish or even offset the mediating effect of frailty on depressive symptoms. Thus, enhancing ADL and acquiring appropriate sleep duration could be beneficial in the alleviation of depression symptoms, especially in the context of frailty and prefrailty.

## Data availability statement

The raw data supporting the conclusions of this article will be made available by the authors, without undue reservation.

## Ethics statement

The studies involving humans were approved by the ethics committee of Zhong Shan Hospital. The studies were conducted in accordance with the local legislation and institutional requirements. The participants provided their written informed consent to participate in this study.

## Author contributions

WS: Investigation, Methodology, Visualization, Writing – original draft, Writing – review & editing, Conceptualization. ML: Investigation, Writing – review & editing, Conceptualization. TY: Data curation, Investigation, Writing – review & editing. DW: Investigation, Writing – review & editing. QY: Investigation, Writing – review & editing. FL: Writing – review & editing, Supervision. QW: Funding acquisition, Writing – review & editing, Conceptualization. YM: Writing – review & editing, Supervision.
