# Supplementary material for: Relationship between frailty and depressive symptoms in older adults: role of activities of daily living and sleep duration
Source: Front Med (Lausanne). 2024 Jun 26;11:1416173. doi: 10.3389/fmed.2024.1416173 (PMC11238213; doi:10.3389/fmed.2024.1416173)
Supplement: Supplementary file 1 [file Data_Sheet_1.docx]

Supplementary Material

# Supplementary Tables

**Supplementary Table 1**

The modified criteria of Physical Frailty Phenotype (PFP)

| Characteristics | Description | |
| --- | --- | --- |
|  | Male | Female |
| Weight loss | Unexplained weight loss of more than 3 kg or more than 5% of body mass in the past one year | |
| Slow pace  (Five-meter walking time) | Height≤173cm：≥7s  Height>173cm：≥6s | Height≤159cm：≥7s  Height>159cm：≥6s |
| Weakness  (Grip strength) | BMI≤24.0kg/m2：≤29 kg  BMI 24.1-26.0kg/m2：≤30 kg  BMI 26.1-28.0kg/m2：≤30 kg  BMI>28.0kg/m2：≤32 kg | BMI ≤23.0kg/m2：≤17 kg  BMI 23.1-26.0 kg/m2：≤17.3 kg  BMI 26.1-29.0 kg/m2：≤18 kg  BMI>29.0 kg/m2：≤21 kg |
| Low physical activity  (Weekly energy expenditure) | <383kcal/week  (About 2.5h walk) | <270kcal/week  (About 2h walk) |
| Exhaustion | According to the Center for Epidemiological Studies—Depression scale (CES-D), self-report of either of: “I felt that everything I did was an effort” and “I could not get going.” were for more than three days and above in the past week | |

BMI, body mass index.

**Supplementary Table 2**

Pearson’s Bivariate Correlation Coefficients between all study variables

| Variables | 1 | 2 | 3 | 4 | 5 | 6 | 7 | 8 | 9 | 10 | 11 | 12 | 13 | 14 | 15 | 16 | 17 | 18 |
| --- | --- | --- | --- | --- | --- | --- | --- | --- | --- | --- | --- | --- | --- | --- | --- | --- | --- | --- |
| 1.Frailty | — |  |  |  |  |  |  |  |  |  |  |  |  |  |  |  |  |  |
| 2.Depressive symptoms | 0.110^**^ | — |  |  |  |  |  |  |  |  |  |  |  |  |  |  |  |  |
| 3.ADL | 0.195^**^ | 0.163^**^ | — |  |  |  |  |  |  |  |  |  |  |  |  |  |  |  |
| 4.Sleep duration | 0.059^*^ | -0.250^**^ | 0.010 | — |  |  |  |  |  |  |  |  |  |  |  |  |  |  |
| 5.Age | 0.287^**^ | 0.039 | 0.141^**^ | 0.067^*^ | — |  |  |  |  |  |  |  |  |  |  |  |  |  |
| 6.Sex | 0.034 | 0.105^**^ | 0.160^**^ | -0.081^**^ | 0.011 | — |  |  |  |  |  |  |  |  |  |  |  |  |
| 7.Education level | -0.134^**^ | -0.032 | -0.136^**^ | -0.027 | -0.073^**^ | -0.458^**^ | — |  |  |  |  |  |  |  |  |  |  |  |
| 8.Marital status | -0.115^**^ | -0.012 | -0.058^*^ | -0.013 | -0.182^**^ | -0.108^**^ | 0.100^**^ | — |  |  |  |  |  |  |  |  |  |  |
| 9.Annual income | -0.094^**^ | -0.035 | -0.062^*^ | -0.018 | -0.069^**^ | -0.195^**^ | 0.307^**^ | 0.052 | — |  |  |  |  |  |  |  |  |  |
| 10.Smoking | -0.047 | -0.057^*^ | -0.120^**^ | 0.035 | -0.066^*^ | -0.555^**^ | 0.207^**^ | 0.018 | 0.117^**^ | — |  |  |  |  |  |  |  |  |
| 11.Drinking | -0.064^*^ | -0.040 | -0.125^**^ | 0.021 | -0.078^**^ | -0.440^**^ | 0.147^**^ | 0.051 | 0.072^**^ | 0.345^**^ | — |  |  |  |  |  |  |  |
| 12.Weekly exercise | -0.241^**^ | -0.079^**^ | -0.028 | 0.009 | 0.062^*^ | -0.026 | 0.097^**^ | -0.016 | 0.041 | -0.046 | 0.003 | — |  |  |  |  |  |  |
| 13.Number of chronic diseases | 0.085^**^ | 0.061^*^ | 0.024 | -0.005 | 0.045 | 0.006 | -0.008 | -0.076^**^ | -0.020 | -0.060^*^ | 0.051 | 0.058^*^ | — |  |  |  |  |  |
| 14.Number of medications | 0.111^**^ | 0.033 | 0.095^**^ | -0.003 | 0.058^*^ | 0.018 | -0.033 | -0.056^*^ | -0.009 | -0.061^*^ | 0.002 | 0.029 | 0.793^**^ | — |  |  |  |  |
| 15.Falls in the last year | 0.065^*^ | 0.034 | 0.049 | -0.070^**^ | 0.014 | 0.086^**^ | -0.080^**^ | -0.017 | -0.037 | -0.074^**^ | -0.033 | -0.040 | 0.043 | 0.114^**^ | — |  |  |  |
| 16.Grip strength | -0.433^**^ | -0.051 | -0.198^**^ | 0.023 | -0.200^**^ | -0.623^**^ | 0.360^**^ | 0.125^**^ | 0.181^**^ | 0.397^**^ | 0.320^**^ | 0.013 | -0.038 | -0.072^**^ | -0.072^**^ | — |  |  |
| 17.Gait speed | -0.560^**^ | -0.044 | -0.196^**^ | -0.050 | -0.295^**^ | -0.178^**^ | 0.210^**^ | 0.110^**^ | 0.127^**^ | 0.144^**^ | 0.114^**^ | 0.081^**^ | -0.121^**^ | -0.147^**^ | -0.101^**^ | 0.296^**^ | — |  |
| 18. BMI | 0.026 | -0.011 | -0.013 | -0.016 | -0.055^*^ | 0.041 | 0.000 | 0.010 | -0.011 | -0.064* | -0.002 | 0.024 | 0.193** | 0.173** | 0.058* | 0.075** | -0.097** | — |

ADL, activities of daily living; BMI, body mass index.

^*^*P* < .05, ^**^P < .01.
